# Effects of Vitamin D on Asprosin Immunoreactivity Against Cyclophosphamide-Induced Liver Injury in Rats

**DOI:** 10.7759/cureus.46711

**Published:** 2023-10-09

**Authors:** Ahmet Turk, Tuncay Kuloglu, Abdullah Karadag, Tuba Ozcan Metin

**Affiliations:** 1 Department of Histology and Embryology, Faculty of Medicine, Adiyaman University, Adiyaman, TUR; 2 Department of Histology and Embryology, Faculty of Medicine, Firat University, Elazig, TUR; 3 Department of Physiology, Faculty of Medicine, Adiyaman University, Adiyaman, TUR; 4 Department of Histology and Embryology, Faculty of Medicine, Kahramanmaras Sutcu Imam University, Kahramanmaras, TUR

**Keywords:** asprosin, cyclophosphamide, liver damage, vitamin d, rat

## Abstract

Background

Cyclophosphamide (CP), commonly used as an anticarcinogenic drug, has the potential to induce detrimental effects on multiple tissues, including the liver. Asprosin, which is a glucogenic adipokine, induces the liver to secrete glucose, thus contributing to the maintenance of homeostasis. This study aims to investigate the immunoreactivity of asprosin in the liver tissue of rats exposed to CP administration, as well as the changes in its levels due to the supplementation of Vitamin D (Vit D).

Materials and methods

Four experimental groups were formed, including control, Vit D (200 IU/kg), CP (200 mg/kg), and Vit D+ CP. Histopathological analysis was carried out by employing staining methods on liver tissues. These techniques encompassed the application of hematoxylin-eosin (H&E), Masson’s trichrome, and periodic acid Schiff (PAS). Through the application of spectrophotometric methods, concentrations of malondialdehyde (MDA), total antioxidant status (TAS), total oxidant status (TOS), and asprosin were determined. Furthermore, apoptotic cells were identified by the terminal deoxynucleotidyl transferase (TdT) dUTP nick-end labeling (TUNEL) method, and the asprosin immunoreactivity was determined by immunohistochemistry.

Results

Under light microscope examination, the histopathological damage was found to be more notable in the CP group compared to the control group. Moreover, a decrease was observed in serum and tissue asprosin levels, while an increase was noted in the count of apoptotic cells, along with elevated MDA and TOS levels. However, in the CP+Vit D group, Vit D administration alleviated histopathological damage. Notably, there were significant increases in TAS and asprosin levels, accompanied by reductions in both MDA and TOS levels.

Conclusions

The effect of CP on liver tissue was observed to result in damage and a reduction in asprosin levels. Vit D supplementation revealed elevated asprosin levels and a distinct protective effect on the tissue. Considering the association between asprosin and liver injury induced by CP, further research is needed to elucidate the mechanisms that underlie the effect of asprosin on tissues. When combined with Vit D, asprosin holds promise for potential clinical applications as a therapeutic target.

## Introduction

A broad spectrum of cancer types, such as lymphoma, breast cancer, and leukemia, is effectively treated using cyclophosphamide (CP). Nonetheless, its utilization is constrained due to severe side effects, such as hepatotoxicity [1,2]. CP’s toxic effects primarily stem from two key metabolites: phosphoramide mustard, the antineoplastic compound, and acrolein, the detrimental agent. Acrolein, characterized by α, β-unsaturated groups, functions as a highly reactive aldehyde and has been recognized as the initiator of lipid peroxidation [3].

The principal factor that restricts the clinical application of CP is exposure to acrolein, which serves as the primary cause of cytotoxicity across all cell types. The principal mechanisms responsible for CP-induced hepatotoxicity involve oxidative stress and the generation of reactive oxygen species (ROS). Moreover, reports indicate that phosphoramides induce apoptosis and DNA damage, similar to the effects of acrolein [4]. Furthermore, increased levels of free radicals damage DNA, activate caspase-3 signaling, and contribute to cellular death [5].

Asprosin is a glucogenic adipokine produced and released by white adipose tissue during periods of fasting by the fibrillin-1 gene [6]. It is recognized for its involvement in numerous metabolic functions, encompassing glucose metabolism, insulin resistance, apoptosis, and the regulation of appetite [7]. The liver emerges as the central focal point for asprosin due to its principal function of storing glycogen and promptly releasing it into the bloodstream during periods of fasting. By binding to receptors on hepatocytes, asprosin facilitates the incorporation of glucose into circulation through on cyclic adenosine monophosphate signaling pathway [8]. Asprosin encompasses a molecule that provides extensive research opportunities in diabetes, obesity, polycystic ovarian syndrome, and cardiovascular disease [7]. In an in vitro study involving mesenchymal stem cells, the researchers concluded that asprosin provided protection against hydrogen peroxide-induced injury and apoptosis. Additionally, it enhanced the functions of these cells and their potential therapeutic effects on myocardial infarction [9].

Although Vit D primarily originates as a prohormone through the synthesis in the epidermis, there has been a global prevalence of its deficiency in recent times, correlating with an elevated susceptibility to specific autoimmune disorders [10]. The efficacy of Vit D extends across diverse physiological pathways, attributed to the widespread presence of Vit D receptor (VDR) in various tissues and organs [11]. Moreover, Vit D assumes diverse functions, serving as an agent with anti-inflammatory and anti-apoptotic activity, while also acting preventively against the onset of cancer, diabetes, cardiovascular, renal, and liver diseases [12,13].

The objective of this research is to examine the serum and tissue levels of asprosin in rat liver tissue treated with CP, along with the alterations in its levels resulting from the administration of Vit D. Although research has explored the impact of CP on liver tissue, there is a lack of evidence concerning their influence on asprosin levels. This study hypothesizes that the combination of Vit D and asprosin may offer potential clinical applications as a therapeutic target.

## Materials and methods

Animals and experimental design

After obtaining approval from the Ethics Committee for Animal Experiments of Fırat University, the research study proceeded as planned (Approval number: 2019/105-172, Date: 09/18/2019). Twenty-eight healthy Wistar albino male rats (aged 8-10 weeks) were divided into four groups, each consisting of seven rats, and were provided ad libitum access to food and water. The environmental temperature was maintained in a constant range of 22 and 25 °C, and monitoring was conducted over a 12-hour light-dark period. The experimental period was designed to last 15 days. The groups were organized as follows: control group (received no treatment); Vit D group (received oral administration of 200 IU/day of Vit D throughout the experimental period, 50.000 IU/15 ml Devit-3 oral drops (DEVA, Istanbul, Turkey) [14]; CP group (received a single intraperitoneal (i.p.) dose of 200 mg/kg CP (endoxan; Eczacıbaşı, Istanbul, Turkey) at the start of the experiment [15], and CP+Vit D group (received a single i.p. dose of 200 mg/kg CP on the first day and 200 IU/day of oral Vit D throughout the experiment).

At the experiment's termination, all rats underwent anesthesia via i.p. injection of ketamine (75 mg/kg) and xylazine (10 mg/kg) anesthesia. Some of the liver tissues were used for biochemical studies, while others were collected in neutral buffered formalin (10%) for light microscopic analysis. Blood samples were collected in serum-separating tubes. After the coagulation process, serum samples were obtained through centrifugation at 3,000 rpm for 10 min and stored at -80 °C until they underwent analysis.

Histopathological analysis

Liver tissues were embedded in paraffin blocks under appropriate conditions, and then it was taken sections of 4-6 μm thickness. Staining techniques including H&E, Masson trichrome, and periodic acid Schiff (PAS) were applied to the sections. The preparations were examined under a Leica DM500 light microscope (Leica Microsystems, Wetzler, Germany) and photographed.

Labeling method (terminal deoxynucleotidyl transferase (TdT) dUTP nick-end labeling (TUNEL))

The identification of cells undergoing apoptosis in the liver was conducted following the manufacturer’s guidelines using the ApopTag Plus Peroxidase In Situ Apoptosis Detection Kit (cat no: S7101, Chemicon International, USA).

At a magnification of 100X, a minimum of 500 normal and apoptotic cells were counted randomly in selected fields for each slide. The statistical assessment encompassed the computation of the apoptotic index (AI), achieved by dividing the count of apoptotic cells by the overall cell count (including both normal and apoptotic cells).

Immunohistochemical staining

Briefly, after applying the block solution to the deparaffinized liver tissues for 5 minutes, they were incubated with an asprosin primary antibody (anti-asprosin antibody, FNab09797, Fine Test, China) for 60 minutes. Then, it underwent a 30-minute incubation with the secondary antibody at room temperature. Following this, it was subjected to a 30-minute incubation with streptavidin peroxidase and then transferred into phosphate-buffered saline (PBS). 3-Amino-9-ethylcarbazole (AEC) substrate along with the AEC chromogen solution was applied onto the sections, followed by counterstaining with Mayer’s hematoxylin. The prepared samples were observed, assessed, and captured in photographs using the Leica DM500 microscope. Histoscore was established based on the prevalence (0.1: <25%, 0.4: 26- 50%, 0.6: 51-75%, 0.9: 76-100%) and severity of immunoreactivity (0: none, +0.5: very little, +1: little, +2: moderate, +3: severe) Histoscore = prevalence x severity [16].

Malondialdehyde (MDA) measurement

Liver tissues were homogenized and centrifuged, and the supernatant was transferred to a separate tube. Moreover, 1 ml of 10% trichloroacetic acid (TCA), 1 ml of 0.6% thiobarbituric acid (TBA), 1 ml of distilled water, and finally 0.5 ml of 4% hydrochloric acid were added to it. After the incubation period, 3 ml of butanol was added to each tube and centrifuged at 5,000 rpm for 5 min. The supernatant fraction was read at 532 nm against butanol in the spectrophotometer. The absorbance value (X) was determined using the formula (X+0.0344)/0.0492).

Total antioxidant status (TAS) and total oxidant status (TOS) measurement

Rel assay brand kit (Rel Assay Kit Diagnostics, Turkey) was used and measured by the manufacturer’s instructions in serum samples. Absorbance values were measured at 532 nm using a spectrophotometer device. TOS and TAS values were expressed as mmol Trolox equivalent/lt and μmol H2O2 equivalent/lt, respectively.

Asprosin measurement

Sunred kit (Shanghai Sunred Biological Technology Co., Ltd. China) was used to measure asprosin levels in serum samples by the enzyme-linked immunosorbent assay (ELISA) method. The experiment procedure was carried out according to the manufacturer’s instructions. Results were obtained as ng/ml.

Statistical analysis

Data analysis was conducted using GraphPad InStat (version 8.01; GraphPad Software, San Diego, CA). The one-way analysis of variance (ANOVA) was used for parametric tests, while the Kruskal-Wallis test was employed for non-parametric tests, followed by a post-hoc Tukey test. The data were presented as median (min-max). P<0.05 was considered statistically significant.

## Results

Histopathological examinations

**Figure 1:**
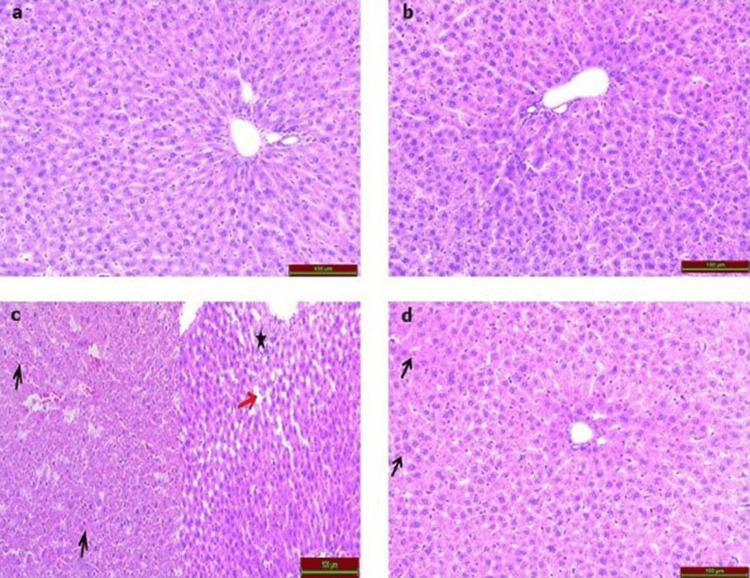
Photomicrographs of H&E-stained rat liver tissues (a) The control and (b) Vit D groups show normal histomorphology. (c) The CP group shows hemorrhage (black arrow), inflammatory cell infiltration (black star), and sinusoidal dilatation (red arrow). (d) The CP+Vit D group shows markedly diminished hemorrhage (black arrow). Scale bar = 100 µm.

The sections of the control (Figure 1a) and Vit D (Figure 1b) groups, stained with H&E revealed that the liver tissue had typical histoarchitecture. The CP group (Figure 1c) exhibited hemorrhage, sinusoidal dilatation, increased glycogen accumulation, and inflammatory cell infiltration while the addition of Vit D (Figure 1d) led to a reduction in histopathological damage. When compared to the control group, the difference in the CP group was statistically significant (p<0.001) (Table 1).

**Table 1:** Analysis of the histopathological scores Values ​​are given as median (min-max). ^a^Compared to the control group, ^b^Compared with the CP group (p<0.01).

	Control Group	Vit D Group	CP Group	CP+Vit D Group
Hemorrhage Median (Min-Max)	0.00 (0.00-1.00)	0.00 (0.00-1.00)	3.00 (2.00-3.00)^a^	0.00 (0.00-1.00)^b^
Inflammatory cell increase Median (Min-Max)	0.00 (0.00-1.00)	0.00 (0.00-1.00)	2.00 (2.00-3.00)^a^	0.00 (0.00-0.00)^b^
Sinusoidal dilation Median (Min-Max)	0.00 (0.00-1.00)	0.00 (0.00-1.00)	2.00 (1.00-3.00)^a^	0.00 (0.00-1.00)^b^
Connective tissue increase Median (Min-Max)	0.00 (0.00-1.00)	0.00 (0.00-1.00)	3.00 (1.00-3.00)^a^	0.00 (0.00-0.00)^b^
Glycogen reduction Median (Min-Max)	0.00 (0.00-1.00)	0.00 (0.00-1.00)	2.00 (1.00-3.00)^a^	0.00 (0.00-0.00)^b^

**Figure 2:**
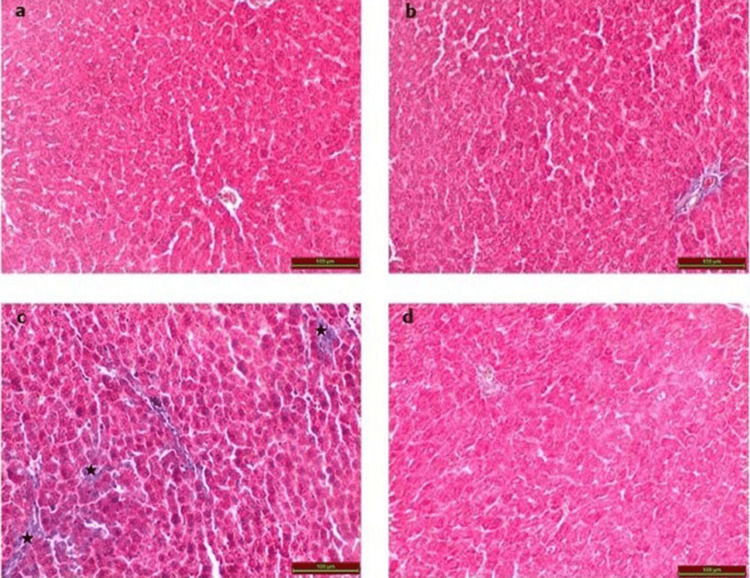
Photomicrographs of Masson’s trichome-stained rat liver tissues (a) Control group; (b) Vit D group; (c) CP group displaying increased connective tissue (black stars); and (d) CP+Vit D group. Scale bar = 100 µm.

Examination of the Masson trichrome-stained sections from the control (Figure 2a) and Vit D (Figure 2b) groups revealed the presence of typical histological architecture. In contrast to the control group, the CP group demonstrated a notable increase in connective tissue (Figure 2c), but the treatment group displayed a decrease in this aspect (Figure 2d, Table 1).

**Figure 3:**
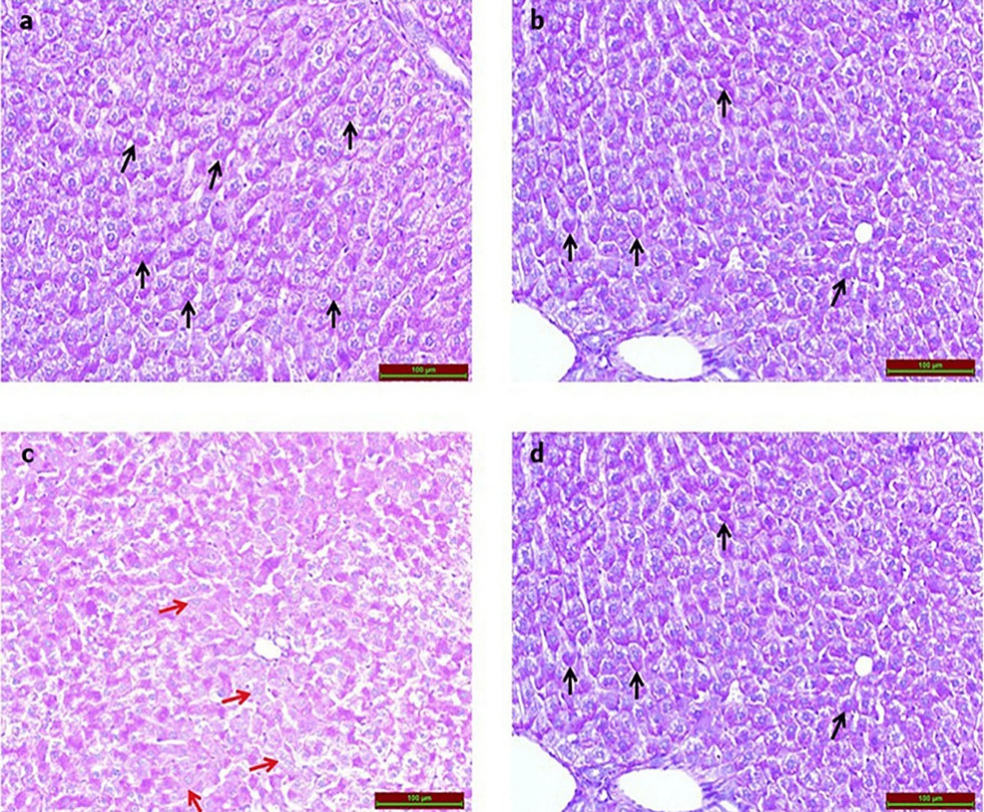
Photomicrographs of the PAS-stained rat liver tissues Glycogen stores were observed at normal levels (black arrows) in (a) the control group, (b) the Vit D group, and (d) the CP+Vit D group. (c) The CP group shows a noticeable increase in glycogen loss in hepatocytes (red arrows). Scale bar 100 = µm.

The PAS-stained sections of the CP group’s sections hepatocytes displayed an increase in glycogen loss in comparison to that of the control group. However, a notable decrease in glycogen loss was evident in the CP+Vit D group (Figure 3, Table 1).

TUNEL results

**Figure 4:**
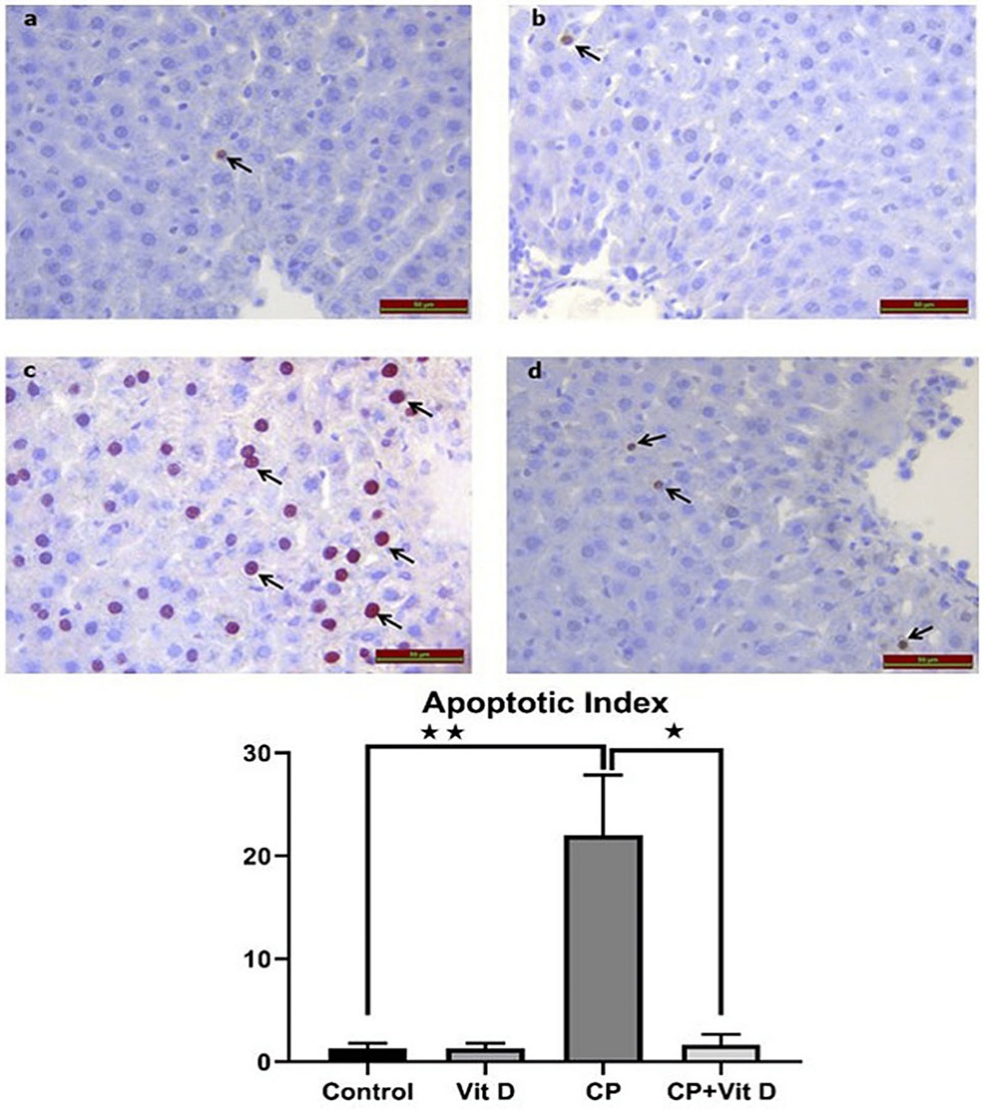
Photomicrographs of the tunnel method (a) Control group; (b) the Vit D group; (c) the CP group shows an increase in the number of apoptotic cells (black arrows); and (d) the CP+Vit D group. The difference between the CP and control groups (**: p<0.001), as well as between the CP and CP+Vit D groups (*: p<0.01) was statistically significant. DAP chromogen, Mayer hematoxylin, scale bar = 50 µm.

Illustrated in Figure 4 are the findings obtained by light microscopy after the utilization of the TUNEL method for staining apoptotic cells. A significant increase was noted in the CP group (Figure 4c) compared to the control group (Figure 4a), as indicated by the apoptotic index analysis (p=0.0072). Moreover, there was a significant decrease observed in the CP+Vit D (Figure 4d) group as compared to the CP group (p<0.0175).

Asprosin immunoreactivity

**Figure 5:**
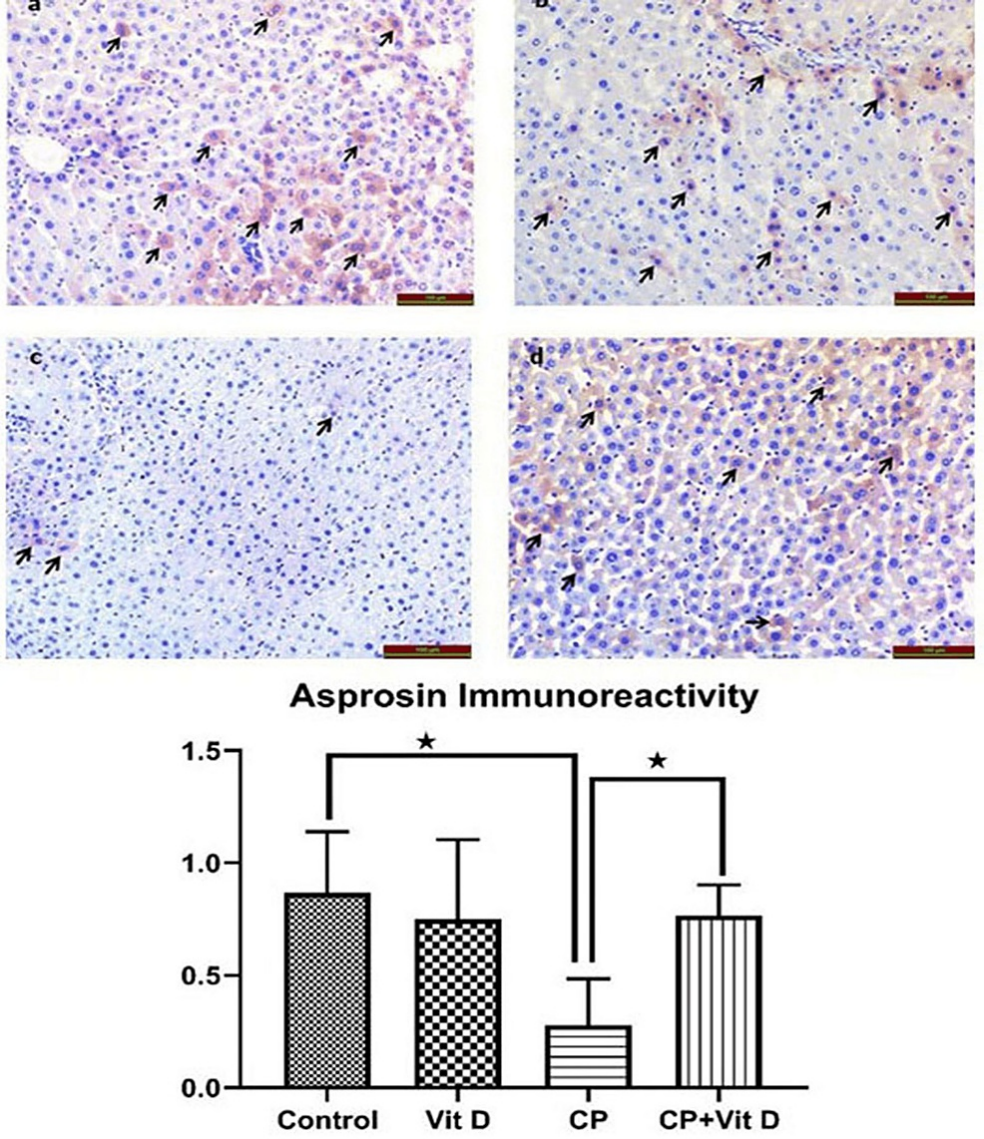
Photomicrographs of the asprosin immunoreactivity in rat liver tissues (a) Control group; (b) the Vit D group; (c) the CP group; and (d) the CP+Vit D group. Asprosin immunoreactive cells are indicated by black arrows. The difference between the CP and Control groups, as well as between the CP and CP+Vit D groups was statistically significant (*: p<0.05). AEC chromogen, Mayer hematoxylin, scale bar = 50 µm.

As shown in Figure 5, there were positive cells in the control (Figure 5a) and Vit D (Figure 5b) groups when the sections with asprosin immunolabeling were examined. A significant decrease in the intensity of asprosin immunoreactivity was observed in the CP group (Figure 5c) when compared to the control group (p=0.0225). The difference between CP and CP+Vit D (Figure 5d) groups was also significant (p=0.0483).

Biochemical results

Malondialdehyde (MDA) Levels

**Figure 6:**
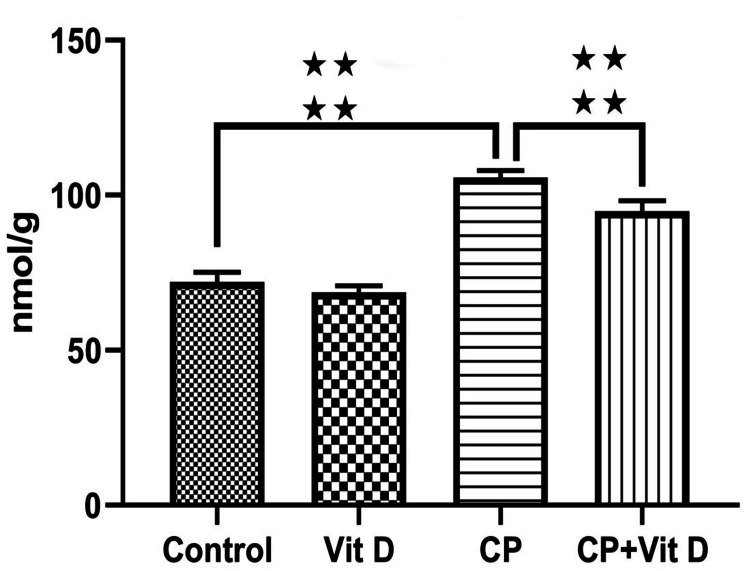
Comparison of the MDA levels of liver tissue between groups The MDA levels were presented as nmol/g. MDA levels exhibited a significant increase in the CP group when compared to the control group (****: p<0.001). MDA levels were significantly decreased in the CP+Vit D group compared to the CP group (****: p<0.001).

Liver MDA levels were similar in the control and Vit D groups (p=0.209). A statistically significant increase was found in the CP group when compared to the control group (p<0.001). In addition, it was observed that the MDA levels of the CP+Vit D group were significantly decreased according to the CP group (p<0.001) (Figure 6).

TAS and TOS levels

**Figure 7:**
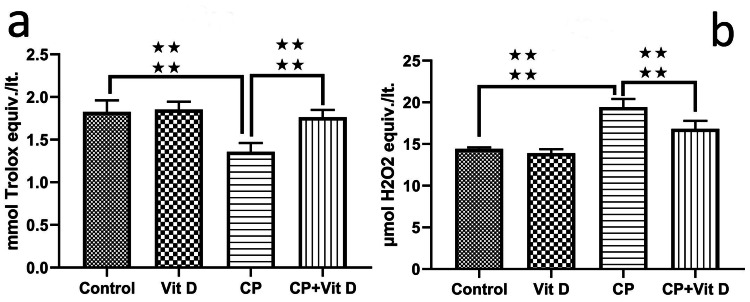
Serum levels of (a) TAS and (b) TOS Results are expressed as mmol Trolox equivalent/lt and as μmol H2O2 equivalent/lt. In comparison to the control group, TAS levels were significantly lower in the CP group (****: p<0.001). There was a notable increase in TAS levels within the CP+Vit D group when compared to the CP group (****: p<0.001). Compared to the control group, TOS levels increased significantly in the CP group (****: p<0.001). Compared to the CP group, TOS levels were significantly decreased in the CP+Vit D group (****: p<0.001).

In comparison to the control group, the CP group has significantly lower TAS levels (p<0.001), while the Vit D group has similar (p=0.805). Notably, the application of Vit D resulted in a significant increase in antioxidant levels when compared to the CP group (p<0.001) (Figure 7a).

Significantly higher levels of TOS were noted in the CP group compared to the control group (p<0.001). Application of Vit D resulted in a significant decrease in oxidant levels compared to the CP group (p<0.001), as illustrated in Figure 7b.

Asprosin levels

**Figure 8:**
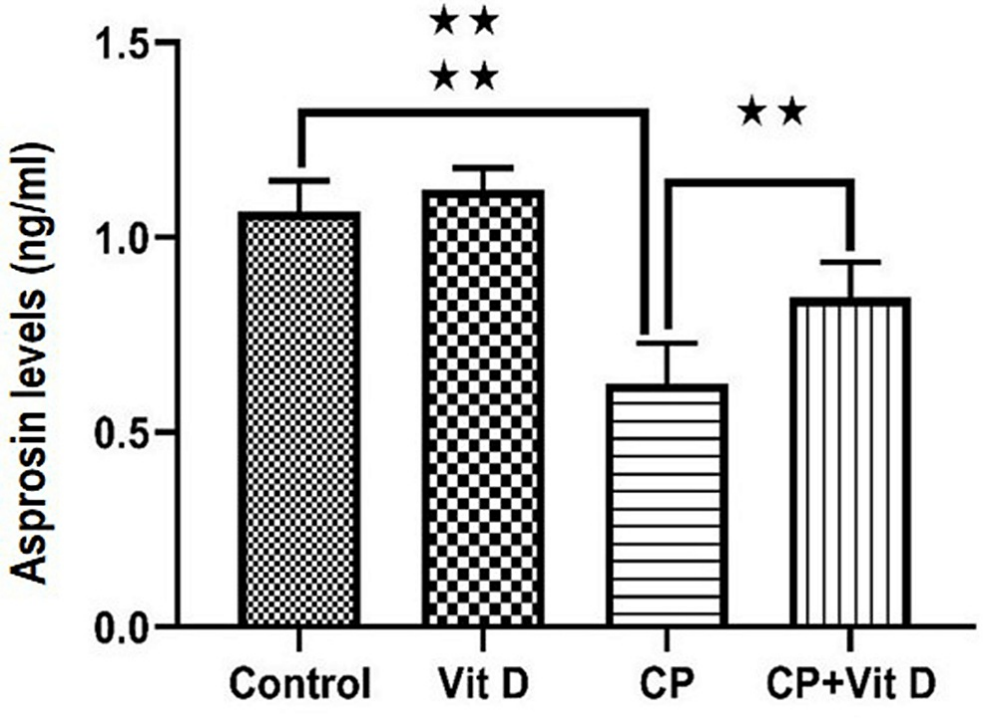
Serum levels of asprosin Asprosin levels are expressed as ng/ml. In comparison to the control group, asprosin levels exhibited a significant decrease in the CP group (****: p<0.0001). In contrast to the CP group, asprosin levels displayed a significant increase in the CP+Vit D group (**: p<0.001).

There were no differences observed between the control and Vit D groups. Notably, asprosin levels exhibited a significant decrease in the CP group in comparison to the control group (p<0.0001), followed by a significant increase after Vit D treatment (p=0.0018) (Figure 8).

## Discussion

Certain antineoplastic agents, such as CP, can lead to some dose-dependent adverse effects on the liver, and, in some cases, these effects can be life-threatening [17]. Along with being toxic, CP elevates the levels of ROS, diminishes tissue antioxidant capacity, and contributes to the buildup of highly reactive oxygen radicals. Consequently, all of these side effects limit its use [18]. In histopathological examinations, it has been reported that CP results in pathological alterations in hepatocytes such as degeneration, vacuolization, necrosis, periportal leukocyte infiltration, sinusoidal dilatation, obstruction, and hemorrhage, as well as an increase in connective tissue [19,20]. Our findings indicate that CP administration leads to sinusoidal dilatation, hemorrhage, inflammatory cell infiltration, and increased connective tissue, accompanied by a reduction in glycogen stores in the hepatocytes. As evidenced by observations, Vit D administration was found to alleviate liver injury and reduce infiltration of inflammatory cells, in agreement with the literature [21,22].

To show the tissue-damaging effects of CP administration, histological staining was carried out on liver tissues, and apoptotic cells were determined using the TUNEL method. In this study, we observed a significant increase in the count of TUNEL-positive cells in the CP group compared to the control. On the other hand, the administration of Vit D led to a reduction in the count of positively stained cells. Both the studies concerning the cytotoxic effects of numerous anticancer agents have revealed that Vit D suppresses pathophysiological cell death pathways in tissues and cells, thereby promoting enhanced cell survival [23,24].

After being released into the bloodstream, asprosin makes its way to the liver. Here, it triggers the liver to produce and release glucose into the blood by engaging with its receptor, olfactory receptor 734. Ultimately, this contributes to the preservation of glucose equilibrium [25]. A reduction in asprosin levels has been documented in the serum, as well as in the liver, kidney, and heart tissues of diabetic rats [26,27]. Consistent with the literature, we observed notable reductions in both asprosin immunoreactivities in tissue and plasma levels among the CP group in comparison to the control group. This decrease might be attributed to a compensatory mechanism involving hyperinsulinemia, which aims to normalize blood glucose levels [7]. Due to liver damage, a decrease in activity is natural, as there will be a reduction in receptor levels. However, following the administration of Vit D, there was a considerable increase in these levels.

MDA, which is one of the main sources of ROS [28], and oxidative stress biomarkers such as TAS and TOS can be used to evaluate the prognosis of diseases [29]. In our study, we observed a notable elevation in MDA and TOS levels in the CP group compared to the control group. Additionally, a decrease in TAS levels was noted. Despite a marked decrease in MDA and TOS following the administration of Vit D, these markers continued to remain elevated compared to the control group. Notably, TAS levels exhibited a significant increase. Similar findings to our results were reported in the previous studies regarding the impact of Vit D exposure on MDA and TOS levels [30]. As observed in this study, Vit D supplementation results in a reduction of ROS formation, an increase in the activity of antioxidant enzymes, and inhibition of lipid peroxidation [30].

An important limitation of the current study is the lack of investigation into the molecular mechanism of the receptor and the associated signaling pathways with which asprosin interacts in liver tissue. Additionally, due to resource limitations, the asprosin levels in the tissue could not be determined using the Western blot method. Given these limitations, we anticipate that future studies will offer a new perspective, potentially enabling researchers to consider asprosin as a therapeutic target when integrated with Vit D in chemotherapeutic regimens.

## Conclusions

Our data reveal that CP causes liver damage and reduces asprosine levels in tissue as observed through immunohistochemistry, as well as in serum as determined by biochemistry. Furthermore, treatment with Vit D was observed to elevate asprosin levels and significantly exert a protective effect on the tissue. Considering the association between asprosin and liver injury induced by CP, further research is needed to elucidate the mechanisms that underlie the effect of asprosin on tissues. When combined with Vit D, asprosin holds promise for potential clinical applications as a therapeutic target.
